# ^18^F-flutemetamol positron emission tomography in cardiac amyloidosis

**DOI:** 10.1007/s12350-020-02363-2

**Published:** 2020-10-06

**Authors:** Maria Papathanasiou, Lukas Kessler, Alexander Carpinteiro, Tim Hagenacker, Felix Nensa, Lale Umutlu, Michael Forsting, Alexandra Brainman, Christoph Kleinschnitz, Gerald Antoch, Ulrich Dührsen, Thomas-Wilfried Schlosser, Ken Herrmann, Tienush Rassaf, Peter Luedike, Christoph Rischpler

**Affiliations:** 1 Department of Cardiology and Vascular Medicine, West German Heart and Vascular Center, University Hospital Essen, Hufelandstrasse 55, 45147 Essen, Germany; 2 Department of Nuclear Medicine, University Hospital Essen, Hufelandstrasse 55, 45147 Essen, Germany; 3 Department of Hematology, West German Tumor Center, University Hospital Essen, Hufelandstrasse 55, 45147 Essen, Germany; 4 Department of Molecular Biology, University of Duisburg-Essen, Hufelandstrasse 55, 45147 Essen, Germany; 5 Department of Neurology, University Hospital Essen, Hufelandstrasse 55, 45147 Essen, Germany; 6 Department of Diagnostic and Interventional Radiology and Neuroradiology, University Hospital Essen, Hufelandstrasse 55, 45147 Essen, Germany; 7 Department of Diagnostic and Interventional Radiology, University Hospital Duesseldorf, Moorenstrasse 5, 40225 Duesseldorf, Germany

**Keywords:** Cardiac amyloidosis, positron emission tomography, amyloid-binding tracers, molecular imaging, transthyretin, light-chain

## Abstract

**Purpose:**

Bone-tracer scintigraphy has an established role in diagnosis of cardiac amyloidosis (CA) as it detects transthyretin amyloidosis (ATTR). Positron emission tomography (PET) with amyloid tracers has shown high sensitivity for detection of both ATTR and light-chain (AL) CA. We aimed to investigate the accuracy of ^18^F-flutemetamol in CA.

**Methods:**

We enrolled patients with CA or non-amyloid heart failure (NA-HF), who underwent cardiac ^18^F-flutemetamol PET/MRI or PET/CT. Myocardial and blood pool standardized tracer uptake values (SUV) were estimated. Late gadolinium enhancement (LGE) and T1 mapping/ extracellular volume (ECV) estimation were performed.

**Results:**

We included 17 patients (12 with CA, 5 with NA-HF). PET/MRI was conducted in 13 patients, while PET/CT was conducted in 4. LGE was detected in 8 of 9 CA patients. Global relaxation time and ECV were higher in CA (1448 vs. 1326, *P* = 0.02 and 58.9 vs. 33.7%, *P* = 0.006, respectively). Positive PET studies were demonstrated in 2 of 12 patients with CA (AL and ATTR). Maximal and mean SUV did not differ between groups (2.21 vs. 1.69, *P* = 0.18 and 1.73 vs. 1.30, *P* = 0.13).

**Conclusion:**

Although protein-independent binding is supported by our results, the diagnostic yield of PET was low. We demonstrate here for the first time the low sensitivity of PET for CA.

**Electronic supplementary material:**

The online version of this article (doi:10.1007/s12350-020-02363-2) contains supplementary material, which is available to authorized users.

## Introduction

Cardiac amyloidosis (CA) is nowadays recognized as a major cause of heart failure (HF) with high mortality rates.^1^ For a long time, CA was considered a rare entity, mainly associated with immunoglobulin light-chain derived amyloid (AL) due to plasma cell dyscrasias. During the recent years, transthyretin amyloidosis (ATTR) was shown to be highly prevalent among older patients with left ventricular hypertrophy, HF with preserved ejection fraction and severe aortic valve stenosis, and represents probably the most underdiagnosed heart disease to date.^2^–^5^ These two distinct disease phenotypes account for the overwhelming majority of CA cases and both are associated with a dismal prognosis. In AL-CA median survival can be < 6 months from the time of diagnosis depending on the severity of cardiac involvement.^6^ ATTR amyloidosis has a longer disease course with a median survival of 3.6 years for the acquired, wild-type disease and varying prognosis for the hereditary type of disease, depending on the underlying transthyretin gene mutation.^7^

The evolution of cardiac imaging techniques has contributed substantially to the raised awareness of the disease. Cardiac magnetic resonance imaging (MRI) is an established diagnostic method for myocardial disease. It enables tissue characterization and facilitates differential diagnosis of myocardial hypertrophy.^8^ Bone-tracer scintigraphy was the first method to enable non-invasive diagnosis of CA. Following the publication of a large-scale study on its diagnostic accuracy compared to endomyocardial biopsy, scintigraphy has played a pivotal role in redirecting the diagnostic pathway from the catheterization laboratory and the pathology bench to the outpatient setting and is now integrated in the diagnostic algorithm for the evaluation of patients with suspected CA.^9^,^10^ Despite this paradigm shift, bone-tracer scintigraphy does not enable non-invasive diagnosis of AL-CA and scarce data exist regarding its diagnostic yield at an early disease stage.

Aiming to fill this gap, positron emission tomography (PET) bears hope for the non-invasive diagnosis of both ATTR- and AL-CA by utilizing tracers with amyloid-specific affinity. Furthermore, due to its superior spatial resolution, quantitative analysis of tracer uptake has the potential to accurately estimate myocardial amyloid burden, facilitate prognosis and assess disease progression and response to therapy. Previous explorative studies demonstrated a high diagnostic accuracy of cardiac PET/CT for the detection of amyloid deposits of both ATTR and AL type in the heart.^11^–^18^ A meta-analysis reported a sensitivity of 95% and a specificity of 98% for the detection of CA.^19^ The aim of the present study was to estimate the diagnostic accuracy of the amyloid-binding tracer ^18^F-flutemetamol in CA by using integrated PET/MR or low-dose PET/CT imaging protocols.

## Methods

### Study Patients and Study Design

We retrospectively included patients who presented at our clinic with suspected cardiac amyloidosis and underwent off-label ^18^F-flutemetamol PET/MRI or low-dose PET/CT as part of a comprehensive diagnostic evaluation for suspected CA. Patients were included in this retrospective analysis only if the diagnosis of CA was confirmed or excluded via established diagnostic methods i.e., myocardial biopsy or the combination of laboratory examinations with bone-tracer scintigraphy. Accordingly, patients were excluded from analysis if the available results could not confirm or exclude CA. To date four PET tracers with amyloid-specific affinity had been approved for brain amyloid imaging: ^18^F-flutemetamol, ^18^F-florbetaben, ^18^F-florbetapir and ^11^C-Pittsburgh compound B. At the time of our PET studies ^18^F-florbetaben, ^18^F-florbetapir and ^11^C-Pittsburgh compound B had been already utilized in studies on CA patients with a meta-analysis of 6 studies and 98 patients reporting a sensitivity of 95% and a specificity of 98% for the detection of CA.^19^ The decision to utilize ^18^F-flutemetamol was made upon local availability, i.e., ^18^F-flutemetamol was the preferred utilized tracer for brain imaging on site. The decision to conduct the PET studies was clinically driven, as an adjunct to other cardiac imaging modalities, i.e., in order to additionally obtain an estimate of the extension of amyloid infiltration. Previously, all patients were informed of the risks and benefits of the procedure and a shared decision was made followed by a written consent to the procedure.

All study participants received a baseline diagnostic evaluation including medical history, physical examination, 12-lead electrocardiogram, a transthoracic echocardiogram and laboratory testing including a work-up for plasma cell proliferative disease (serum-free light-chain assay, serum and urine immunofixation and serum protein electrophoresis) as part of a standardized procedure at our institution. Depending on the clinical context, additional procedures were performed in order to confirm or rule out the diagnosis of CA. These included endomyocardial biopsy, whole-body planar scintigraphy 3 h after intravenous injection of ^99m^Tc labeled 3,3-diphosphono-1,2-propanodicarboxylic acid (^99m^Tc-DPD), cardiac MRI with late gadolinium enhancement (LGE) and T1 mapping and transthyretin gene sequencing for amyloidogenic variants.

The diagnosis of CA relied upon one of the following:

- Histological confirmation of amyloid deposits on endomyocardial biopsy
- Positive ^99m^Tc-DPD scintigraphy (Perugini stage 2 or 3) in the absence of laboratory indices of AL amyloidosis (monoclonal gammopathy in serum immunofixation or urine immunofixation, elevated free light chains or free light-chain ratio) according to current practice recommendations.^20^,^21^
- Cardiac MRI with typical findings of CA in a patient with previously diagnosed hereditary ATTR polyneuropathy with genetic confirmation of an amyloidogenic transthyretin mutation (hereditary ATTR amyloidosis).

Patients underwent simultaneous cardiac PET/MRI in an integrated system with 3 Tesla field strength according to the examination protocol described below. In case of a non-conditional implanted cardiac electronic device, low-dose PET/computed tomography (PET/CT) was performed for attenuation correction instead of PET/MRI. The study was approved by the local institutional review board for retrospective data analysis (registry number: 19-5786-BO). All study procedures were in accordance with the institutional ethical standards and the declaration of Helsinki.

### PET Protocol and Image Analysis

Scans were performed on an integrated whole-body PET/MRI system (Biograph mMR, Siemens Healthcare, Erlangen, Germany) or on state-of-the-art PET/CT systems (Siemens Biograph mCT or Siemens Biograph Vision, Siemens Healthcare, Erlangen, Germany). A mean activity of 182.1 ± 18.5 MBq of ^18^F-flutemetamol (Vizamyl ^TM^, GE Healthcare, Medi-Physics, Inc., Arlington Heights, IL 60004 USA) was administered intravenously as previously described.^12^,^22^,^23^ Acquisition timepoints were chosen approximately 60 to 90 minutes. p.i. according to the label of ^18^F-flutemetamol. The PET scan started 77.8 ± 18.7 minutes after tracer injection and comprised a cardiac PET scan with 1 bed position and 3D image reconstruction (2 × 2 × 2 mm voxel size) using ordinary Poisson ordered subset expectation maximization with 3 iterations and 21 subsets, a Gaussian filter with 5.0 mm full-width at half-maximum and a 344 × 344 image matrix.^23^ For automatic attenuation correction of the acquired PET data, a four-compartment model attenuation map was calculated from fat-only and water-only Dixon-based sequences by segmentation into background, lung, fat, and soft tissue. In a subgroup of patients (n = 5) dynamic acquisition over 30 minutes was performed with the PET list-mode acquisition starting simultaneously with tracer injection as several previous studies have shown that this scan time is sufficient.^13^,^22^,^24^

List-mode data were reconstructed into the following frames: 12 frames with 5 seconds each, 4 frames with 30 seconds each, 7 frames with 60 seconds each and 4 frames of 300 seconds each. The estimated whole-body effective radiation dose to each patient was 5.82 mSv. Left ventricular myocardial and blood pool standardized uptake values (SUV) were determined and normalized to bodyweight. On the short-axis DICOM reconstructed images of the left ventricular myocardial boundaries were traced over the entire LV myocardial volume at the base, mid ventricle and apex (except for the LV apex segment). A region of interest (ROI) in the center of the left ventricle near the base was used to determine the blood pool SUVs. Scans were visually inspected and then reported as positive if myocardial tracer uptake was visually higher than the background uptake. There were no borderline or equivocal findings in our patient cohort. The myocardial retention index was calculated for the subgroup of patients with dynamic PET studies as previously described.^13^ Briefly, the mean left ventricular myocardial radiotracer uptake between 10 and 30 minutes after ^18^F-flutemetamol injection was divided by the integral of the blood pool time-activity curve from 0 to 20 minutes after tracer injection.

### MRI Protocol and Image Analysis

Cardiac MRI was performed simultaneously with PET on an integrated whole-body 3 Tesla PET/MRI system (Biograph mMR, Siemens Healthcare, Erlangen, Germany). T1 mapping before and after administration of contrast as well as LGE study were performed. For T1 mapping, basal and midventricular short-axis and 4-chamber long-axis images were acquired by the modified Look-Locker inversion recovery (MOLLI). After the standard LGE imaging the T1 measurement was repeated with the MOLLI sequence. T1 measurement was performed by drawing a region of interest in the basal to mid-septum of the appropriate 4-chamber map. For extracellular volume (ECV) measurement, a single region of interest was drawn in each of the 4 required areas: myocardial T1 estimates (basal to mid-septum in 4-chamber map) and blood T1 estimates (left ventricular cavity blood pool in 4-chamber map) before and after contrast administration. Hematocrit was available in all subjects. ECV was calculated according to the established formula: myocardial ECV = (1 − hematocrit) (Δ*R*1_myocardium_/Δ*R*1_blood_), where *R*1 = 1/*T*1.

### Statistical Analysis

Continuous variables are summarized as means (standard deviations), unless indicated otherwise, and categorical variables as counts (percentages). Continuous data were evaluated for normality of distribution with the Shapiro–Wilk test. The two-sided *t* test was used for comparison of continuous, normally distributed data, otherwise the non-parametric Mann–Whitney U-test. The *x*^2^ test or the Fischer’s exact test was used for testing the association between two categorical variables. The level of significance was set at 0.05. All analyses were performed using SPSS (IBM Corp., SPSS Statistics, version 23.0. Armonk, NY).

## Results

**Fig. 1:**
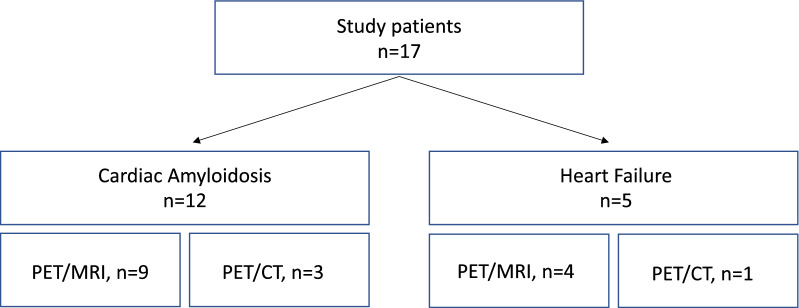
Diagram depicting the imaging modality used in the amyloidosis and heart failure groups (^18^F-flutemetamol PET/MRI or ^18^F-flutemetamol PET/CT)

Our analysis included 17 patients who underwent cardiac ^18^F-flutemetamol PET imaging, 12 patients with CA and 5 with NA-HF. The study patients received an integrated ^18^F-flutemetamol PET/MR (n = 13) or a low-dose ^18^F-flutemetamol PET/CT (n = 4). The selection of the imaging method is demonstrated in Figure 1. ^18^F-flutemetamol PET/MRI was the preferred imaging method and was substituted by ^18^F-flutemetamol PET/CT due to non-conditional implanted devices in 4 patients. Among the amyloidosis patients, 7 were diagnosed with wild-type ATTR-CA, 3 with hereditary ATTR-CA and 2 with AL-CA. The following pathologic ATTR variants were found by gene sequencing in the patients with hereditary disease: p.Val142Ile, p.Glu81Lys and p.Ile127Val. At the time of the PET scan 3 patients were on treatment: two patients with hereditary ATTR-CA received due to preceding ATTR polyneuropathy tafamidis for 24 months and patisiran for 4 months, respectively, and one patient with AL amyloidosis had completed 6 cycles of chemotherapy with bortezomib, cyclophosphamide, and dexamethasone and was in partial remission, according to hematological response criteria.^25^ The baseline characteristics of the amyloidosis group and the HF group are shown in Table 1.

**Table 1:** Baseline characteristics

	Amyloidosis n = 12	NA-HF n = 5	*P* value
Age (years)	71.3 ± 9.5	71.2 ± 8.8	0.99
Male Gender	11 (91.6)	4 (80)	0.52
Race			0.71
Caucasian	11 (91.7)	5 (100)	
African	1 (8.3)	0 (0)	
BMI (kg/m^2^)	24.4 ± 4.3	27.4 ± 5.9	0.26
Amyloid Type			
Wild-type ATTR	7 (58.3)	–	
Hereditary ATTR	3 (25.0)	–	
AL	2 (16.7)	–	
NYHA class			
I	0 (0.0)	1 (20)	0.29
II	4 (33.3)	2 (40)	0.61
III	8 (66.6)	2 (40)	0.59
NT-proBNP (pg/mL)*	2719 (444–10599)	2763 (49–8939)	0.56
eGFR (mL/min/1.73 m^2^)	61.9 ± 13.5	51.9 ± 14.7	0.19

*AL*, Light-chain amyloid; *ATTR*, transthyretin amyloid; *BMI*, Body Mass Index; *eGFR*, estimated glomerular filtration rate; *NT-proBNP*, N-terminal-pro B-type natriuretic peptide; *NYHA*, New York Heart Association. *The given values represent median (min-max)

Morphological and hemodynamic assessment by echocardiography revealed typical features of infiltrative cardiomyopathy among patients with confirmed CA. Mean left ventricular ejection fraction was preserved. Left and right ventricular hypertrophy as well as a higher grade of diastolic dysfunction were observed in the amyloidosis group, as opposed to the group of NA-HF (Table 2). Cardiac MRI was conducted in 9 of 12 amyloidosis patients and 4 of 5 patients of the NA-HF group, as shown in Figure 1. LGE of a diffuse, subendocardial or transmural pattern was detected in 8 out of 9 amyloidosis subjects. In one patient with biopsy-proven wild-type ATTR-CA no LGE was found. Transmural, infarct-associated LGE was shown in 1 of the 4 patients in the HF group. Global relaxation time by native T1 mapping was overall prolonged and significantly longer in CA patients. Mean ECV was profoundly increased (58%) among CA patients, which is a common finding of the disease.^26^

**Table 2:** Imaging data

	Amyloidosis n = 12	NA Failure n = 5	*P* value
Echocardiography			
LVEF (%)	53.8 ± 10.6	49.4 ± 8.9	0.43
LVMM index (g/m^2^)	201.6 ± 47.4	132.2 ± 24.5	**0.008**
IVSd (mm)	20.5 ± 4.6	12.2 ± 1.8	**0.002**
Left Atrial Volume Index (g/m^2^)	47.2 ± 20.1	58.8 ± 24.6	0.32
PASP (mmHg)	42.2 ± 13.0	42.0 ± 11.5	0.98
*E*′ (cm/s)	6.1 ± 1.9	9.3 ± 4.1	**0.04**
*E*/*E*′	13.9 ± 3.6	12.4 ± 5.2	0.51
Right ventricular hypertrophy	10 (83.3)	0 (0.0)	**0.003**
PET			
SUV max	2.21 ± 0.99	1.69 ± 0.51	0.18
SUV mean	1.73 ± 0.76	1.30 ± 0.33	0.13
TBR max	1.34 ± 0.84	0.92 ± 0.09	0.11
TBR mean	1.36 ± 0.83	0.95 ± 0.11	0.12
MRT*			
LVEF (%)	52.6 ± 13.5	48.0 ± 25.8	0.68
LVMM index (g/m^2^)	228.6 ± 66.3	139.0 ± 43.5	**0.04**
Global relaxation time (ms)	1448 ± 53.1	1326 ± 57.4	**0.02**
Extracellular volume (%)	58.9 ± 14.2	33.7 ± 5.1	**0.006**
Late gadolinium enhancement	8 (88.9)	1 (25.0)	0.05

E′ = Peak velocity of early diastolic mitral annular motion, E/E′ = Ratio of peak velocity of early diastolic transmitral flow to peak velocity of early diastolic mitral annular motion, IVSd = End diastolic Diameter of the Intraventricular Septum, *LVEF*, left ventricular ejection fraction; *LVMM*, left ventricular muscle mass; *PASP*, pulmonary artery systolic pressure; *SUV*, standardized uptake value; *TBR*, target-to-background ratio

*MR was conducted in 9 of 12 amyloidosis patients and 4 of 5 patients of the comparison group

**Fig. 2:**
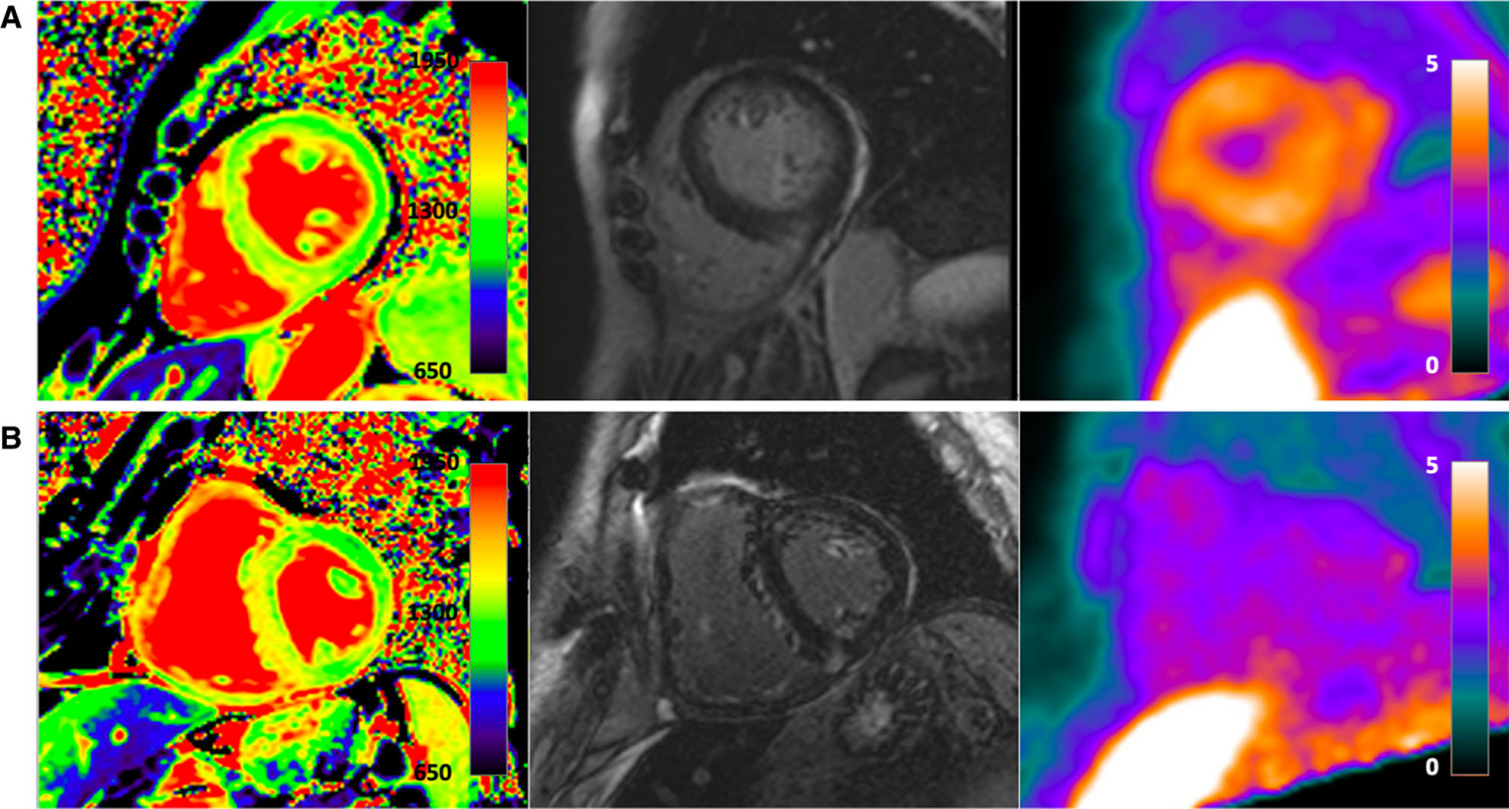
^18^F-flutemetamol PET/MRIs of a PET-positive, female patient with hereditary ATTR-CA (**A**) and a PET-negative male patient with wild-type ATTR-CA (**B**). Representative short-axis images of native T1 maps (left column), late gadolinium enhancement (middle column), and F-18 flutemetamol PET (right column). While patient A demonstrates an abnormal T1 map (1448 ms), diffused LGE, and an increased tracer uptake (SUV_mean_ myocardium: 3.1, SUV_mean_ blood pool: 1.3), patient B does not demonstrate an increased tracer uptake (SUV_mean_ myocardium 1.4, SUV_mean_ blood pool: 1.5) despite an abnormal native T1 map (1460 ms), focal areas of LGE, and a positive myocardial biopsy and bone scan (Perugini 3, not shown)

Visual assessment of the acquired PET images revealed an increased uptake in 2 of 12 amyloidosis patients (one patient with AL-CA under chemotherapy and one with hereditary ATTR-CA under treatment with Patisiran) and in none of the patients in the NA-HF group. Figure 2 demonstrates representative findings of a true-positive and a false-negative PET/MR study. Maximal and mean standardized uptake values (SUV), as well as maximal and mean target-to-background (TBR) ratio did not differ significantly between the two groups (SUVmax 2.2 ± 1.0 vs. 1.7 ± 0.5, *P* = 0.18; SUVmean 1.7 ± 0.8 vs. 1.3 ± 0.3, *P* = 0.13; TBRmax 1.3 ± 0.8 vs. 0.9 ± 0.1, *P* = 0.11; TBRmean 1.4 ± 0.8 vs. 0.9 ± 0.1, *P* = 0.11). Thus, a high proportion of false-negative PET results with an overall low sensitivity of 16.7% was observed in the present cohort. The quantitative assessment of tracer uptake for the two groups is shown in Table 2. In the Supplementary Table 3, we present clinical data of the individual amyloidosis patients and the corresponding PET results.

**Table 3:** Clinical characteristics of patients with confirmed cardiac amyloidosis

Patient	Disease type	Sex	Age	NYHA FC	Race	Symptoms
#1	wtATTR-CA	Male	77	III	Caucasian	Dyspnea, pericardial effusion
#2	wtATTR-CA	Male	64	II	Caucasian	Dyspnea
#3	wtATTR-CA	Male	80	III	Caucasian	Dyspnea
#4	wtATTR-CA	Male	79	III	Caucasian	Dyspnea
#5	wtATTR-CA	Male	77	III	Caucasian	Dyspnea
#6	wtATTR-CA	Male	81	III	Caucasian	Dyspnea
#7	wtATTR-CA	Male	82	III	Caucasian	Dyspnea
#8	hATTR-CA	Female	67	III	Caucasian	Dyspnea, Kachexia
#9	hATTR-CA	Male	73	II	Caucasian	Dyspnea
#10	hATTR-CA	Male	56	III	African	Dyspnea
#11	AL-CA	Male	58	III	Caucasian	Dyspnea
#12	AL-CA	Male	61	III	Caucasian	Dyspnea

Patient	NT-proBNP (pg/ml)	Organ involvement	Therapy	Dx confirmed via	PET	Time p.i.(min)
#1	3661	Heart	–	EMB	Negative	75
#2	2085	Heart	–	EMB	Negative	40
#3	10599	Heart	–	EMB	Negative	60
#4	10513	Heart	–	EMB	Negative	60
#5	1070	Heart	–	SPECT	Negative	81
#6	2775	Heart	–	EMB	Negative	59
#7	7441	Heart	–	SPECT	Negative	101
#8	848	Heart, peripheral and autonomic nervous system	Patisiran	MRI, Gene sequencing	Positive	88
#9	444	Heart, peripheral and autonomic nervous system	Tafamidis	EMB	negative	60
#10	2663	Heart, peripheral and autonomic nervous system	–	MRI, Gene sequencing	Negative	60
#11	5559	Heart, kidney, liver	Chemotherapy	EMB	Positive	88
#12	1816	Heart, kidney	–	EMB	Negative	60

**Fig. 3:**
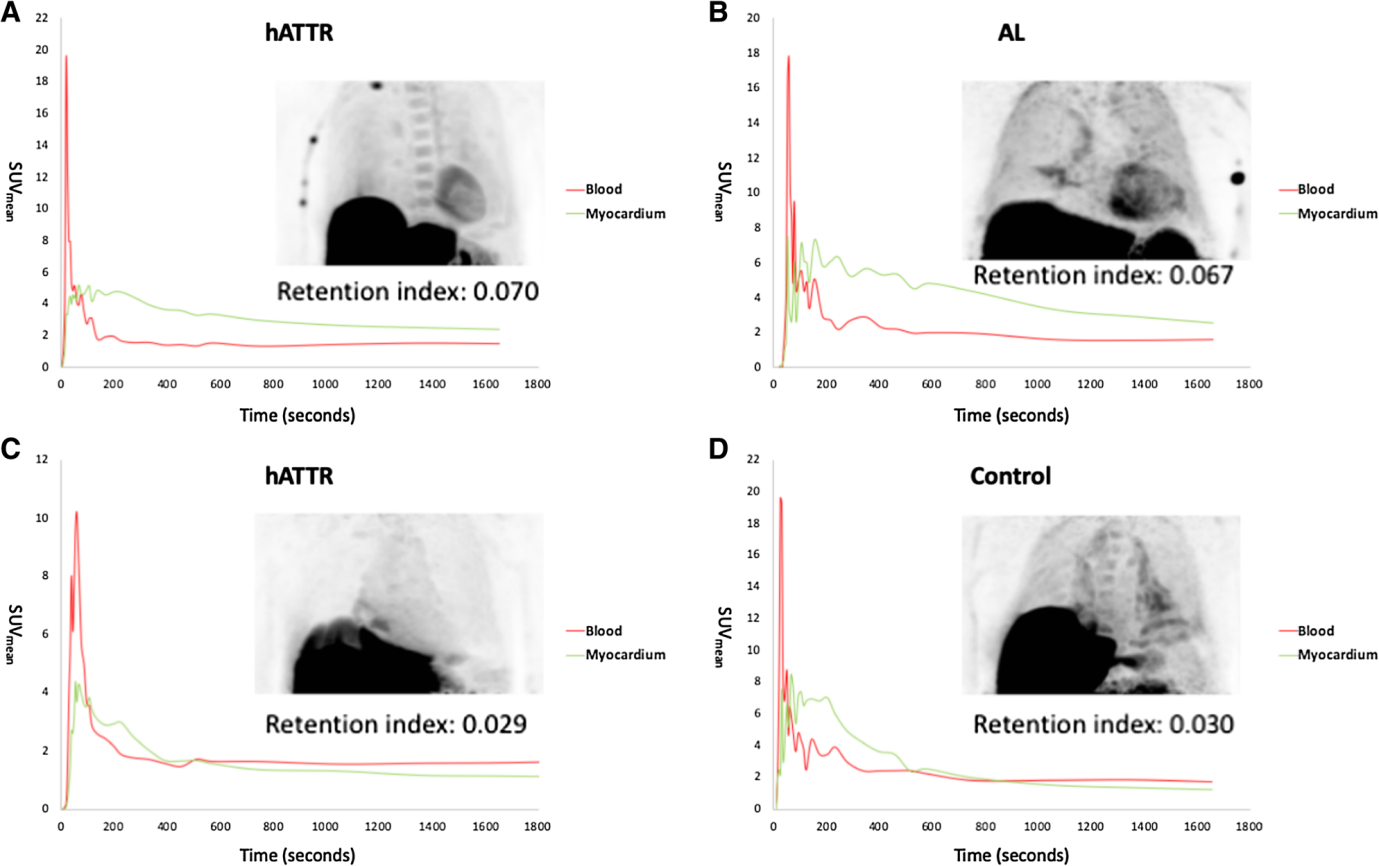
Tracer kinetics in the left ventricular myocardium and the blood. SUVs of the left ventricular myocardium and blood pool are depicted in PET-positive hereditary ATTR-CA (**A**), PET-positive AL-CA (**B**), PET-negative hereditary ATTR-CA with positive endomyocardial biopsy (**C**), and a healthy control (**D**). In the first few minutes, the mean left ventricular and blood pool SUVs are similar for all patients. While the SUVs of the left ventricular myocardium then remain above the SUVs of the blood pool in the PET-positive hereditary ATTR-CA (**A**) and AL-CA patients (**B**), the SUVs of the myocardium decrease in the PET-negative hereditary ATTR-CA (**C**) and the healthy control (**D**) and even fall below the SUVs of the blood pool after approximately 400-600 seconds. Unspecific pericardial tracer uptake can be seen in all patients

Figure 3 demonstrates representative findings in the subgroup of patients with dynamic PET acquisition according to the aforementioned protocol. In panels A and B positive PET studies from two patients with hATTR-CA and AL-CA are depicted. The calculated retention index was 0.072 and 0.067. However, in patients with CA and false-negative PET studies as well as in patients with NA-HF, we found an early tracer accumulation in the left ventricular myocardium which disappeared after a few minutes (green curve) indicating a perfusion-dependent, non-specific tracer accumulation early after tracer injection, as shown in Figure 3, panels C-D. The calculated retention index ranged between 0.027 and 0.030 among patients with negative PET studies.

## Discussion

Radionuclide imaging with bone-avid tracers has emerged as a valuable diagnostic tool for the diagnosis of CA and has even replaced endomyocardial biopsy in certain clinical scenarios. Although the mechanism of tracer uptake and the selectivity of bone-avid tracers for ATTR are not completely understood, their accuracy has been well described.^27^ The method is widely available at a reasonable cost, but its value is confined to ATTR-CA. Detection of both AL and ATTR-CA with ^18^F- and ^11^C-labeled radiotracers with amyloid-specific affinity has already been shown feasible. These positron emitting radiotracers firstly received approval in 2012 for beta-amyloid imaging in the brain to differentiate Alzheimer’s disease from other dementias. Subsequently, a series of investigations revealed a very high accuracy of ^18^F-florbetapir, ^18^F-florbetapen and ^11^C-Pittsburgh compound B for the detection of amyloid in the heart.^11^–^18^ Notably, AL patients exhibited a higher myocardial tracer activity than ATTR, indicating a different binding mechanism and distinct biologic properties of ATTR and AL amyloid. All studies included small study samples; however, according to a meta-analysis including six studies and 98 patients in total, 95% sensitivity and 98% specificity were calculated.^19^

The present study on the utility of ^18^F-flutemetamol in CA is the first to demonstrate that an amyloid-binding PET tracer exhibits a low sensitivity for the detection of amyloid in the heart. Despite a uniform study protocol only two patients, who both received a PET/MRI examination and were both under anti-amyloid treatment, showed increased myocardial uptake. Both patients had a typical distribution, with both atrial and ventricular involvement, as expected in CA. Although the positive scans originate from patients with AL-CA and hereditary ATTR-CA, which supports the aspect of protein-independent tracer binding, the majority of patients had negative PET scans. There were no borderline or equivocal results in this study. Based on the tracer kinetics that were shown here in subgroup of patients with dynamic measurements, we suggest that the early tracer accumulation and decay is a perfusion-dependent and tracer-specific phenomenon, which needs to be further elucidated in the preclinical setting, e.g., in the context of an autoradiography study, as previously described by other groups.^22^

The first study that performed ^18^F-flutemetamol PET/CT in cardiac amyloidosis demonstrated positive results in 8 out of 9 patients with CA.^12^ In this study image acquisition took place much earlier than in our study, between the 10th and 30th minute post injection. This may explain the higher number of positive results (8 out of 9 patients with positive PET studies), as tracer decay at a level lower than that of the blood pool was observed at 77.8 ± 18.7 minutes post injection in our study. While in our study imaging was performed relatively late after tracer injection (apart from the subgroup with additional dynamic acquisition), it is important to note that in previously published studies, a late acquisition was still able to allow the detection of cardiac amyloidosis with high accuracy so that this cannot be the reason for the observed low sensitivity.^13^,^22^

Recently, Möckelind et al,^22^ performed cardiac ^18^F-flutemetamol PET/CT in a cohort consisting of 21 patients with ATTR polyneuropathy carrying a specific ATTR mutation (Val30Met) and 6 controls. All 21 patients were previously treated with liver transplantation or medical therapy. None of the patients included in the study had previously undergone endomyocardial biopsy. Out of 13 patients with available DPD scintigraphy only one was positive, thus at least 12 of 21 patients included in this study did not have a CA according to current diagnostic criteria. This is supported by the echocardiographic findings of the study (e.g., mean interventricular septal diameter was 11 mm), whereas cardiac MRI studies were not available in order to support the diagnosis of CA. The authors calculated an SUV cut-off of 1.46 for the interventricular septum at 30 minutes and defined values above this threshold as positive. Accordingly, the study reported 17 positive PET studies. However, 4 PET studies were negative including the only patient with positive DPD scintigraphy and LV hypertrophy. The authors hypothesize that the negative PET studies may result from the type of amyloid fibrils, as type A amyloid fibrils were previously shown to have lower uptake of ^11^C-PIB and may also have altered perfusion properties. However, in the absence of confirmation or rule out of CA, these findings are very difficult to interpret. Interestingly, the authors observed a very early decay in the dynamic studies of PET-negative patients similar to our findings in patients with confirmed CA and false-negative PET.

Despite being contradictory to the results of other studies, our findings raise questions regarding the ubiquity and generalizability of the available evidence. At least one of 9 patients with confirmed CA in one ^18^F-flutemetamol study^12^ and 1 of 12 patients in another study^14^ utilizing ^11^C-Pittsburgh compound B had a false-negative PET scan. In the small series available to date, this fact points out at a missing piece of the puzzle. Larger studies and experimental evidence regarding the underlying molecular mechanisms of amyloid binding are still needed in order to better define the accuracy of the available amyloid-binding PET tracers.

The present study has the inherent limitations of a retrospective, single-center study with a small sample size. Since there are no comparative data with different tracers in the same population, these results cannot be extrapolated to other available amyloid-binding tracers. Due to the limited number of positive PET studies, a comparative analysis between ECV and tracer uptake as markers of the total amyloid burden in the myocardium was not feasible. Furthermore, dynamic PET acquisition was performed only in a subgroup of patients. PET readers were blinded for clinical data but due to concurrent PET and MRI acquisition in most of the cases the readers were aware of the likelihood of CA based on the characteristic MRI findings in amyloidosis. However, no borderline findings were reported in this study.

According to our results ^18^F-flutemetamol is the first amyloid-specific tracer that exhibits a low sensitivity for CA. Amyloid-binding may be impeded by tracer- and patient- or amyloid-specific factors. A robust imaging methodology is still challenging. Further studies need to investigate the potential limitations of molecular imaging in amyloidosis in order to provide sufficient evidence and guidance for future clinical implementation.

## New Knowledge Gained

In contrast to other available amyloid PET tracers the sensitivity of ^18^F-Flutemetamol for the diagnosis of cardiac amyloidosis was low > 30 minutes post injection. The appropriate imaging protocol for this specific radiotracer still needs to be defined, as tracer kinetics in myocardium and biologic properties of amyloid deposits may influence its diagnostic accuracy.

## Electronic supplementary material

Below is the link to the electronic supplementary material.

Electronic supplementary material 1 (PPTX 4492 kb)

Electronic supplementary material 2 (M4A 7832 kb)

## Abbreviations

ATTR: Transthyretin amyloidosis
AL: Light-chain amyloidosis
CA: Cardiac amyloidosis
ECV: Extracellular volume
LGE: Late gadolinium enhancement
NA-HF: Non-amyloid heart failure
PET: Positron emission tomography
PET/CT: Positron emission tomography /computed tomography
SUV: Standardized uptake values
^99m^Tc-DPD: 3,3-Diphosphono-1,2-propanodicarboxylic acid

## References

[1] Seferović PM, Polovina M, Bauersachs J (2019). Heart failure in cardiomyopathies: a position paper from the Heart Failure Association of the European Society of Cardiology. Eur J Heart Fail..

[2] Mohammed SF, Mirzoyev SA, Edwards WD (2014). Left ventricular amyloid deposition inpatients with heart failure and preserved ejection fraction. JACC Hear Fail..

[3] González-López E, Gallego-Delgado M, Guzzo-Merello G (2015). Wild-type transthyretin amyloidosis as a cause of heart failure with preserved ejection fraction. Eur Heart J..

[4] Treibel TA, Fontana M, Gilbertson JA (2016). Occult transthyretin cardiac amyloid in severe calcific aortic stenosis. Circ Cardiovasc Imaging..

[5] Scully PR, Treibel TA, Fontana M (2018). Prevalence of cardiac amyloidosis in patients referred for transcatheter aortic valve replacement. J Am Coll Cardiol..

[6] Gertz MA (2018). Immunoglobulin light chain amyloidosis diagnosis and treatment algorithm 2018. Blood Cancer J..

[7] Maurer MS, Hanna M, Grogan M (2016). Genotype and phenotype of transthyretin cardiac amyloidosis: THAOS (Transthyretin Amyloid Outcome Survey). J Am Coll Cardiol..

[8] Martinez-Naharro A, Treibel TA, Abdel-Gadir A (2017). Magnetic resonance in transthyretin cardiac amyloidosis. J Am Coll Cardiol..

[9] Dorbala S, Ando Y, Bokhari S (2019). Expert consensus recommendations. J Nucl Cardiol..

[10] Dorbala S, Ando Y, Bokhari S (2019). Expert consensus recommendations. J Nucl Cardiol..

[11] Antoni G, Lubberink M, Estrada S (2013). In vivo visualization of amyloid deposits in the heart with 11C-PIB and PET. J Nucl Med..

[12] Dietemann S, Nkoulou R (2019). Amyloid PET imaging in cardiac amyloidosis: a pilot study using 18F-flutemetamol positron emission tomography. Ann Nucl Med..

[13] Dorbala S, Vangala D, Semer J (2014). Imaging cardiac amyloidosis: a pilot study using 18 F-florbetapir positron emission tomography. Eur J Nucl Med Mol Imaging..

[14] Ezawa N, Katoh N, Oguchi K, Yoshinaga T, Yazaki M, Sekijima Y (2018). Visualization of multiple organ amyloid involvement in systemic amyloidosis using 11C-PiB PET imaging. Eur J Nucl Med Mol Imaging..

[15] Law WP, Wang WYS, Moore PT, Mollee PN, Ng ACT (2016). Cardiac amyloid imaging with 18F-florbetaben PET: a pilot study. J Nucl Med..

[16] Lee SP, Lee ES, Choi H (2015). 11C-Pittsburgh B PET imaging in cardiac amyloidosis. JACC Cardiovasc Imaging..

[17] Osborne DR, Acuff SN, Stuckey A, Wall JS (2015). A routine PET/CT protocol with streamlined calculations for assessing cardiac amyloidosis using 18F-florbetapir. Front Cardiovasc Med..

[18] Pilebro B, Arvidsson S, Lindqvist P (2018). Positron emission tomography (PET) utilizing Pittsburgh compound B (PIB) for detection of amyloid heart deposits in hereditary transthyretin amyloidosis (ATTR). J Nucl Cardiol..

[19] Kim YJ, Ha S, Kim Y (2020). Cardiac amyloidosis imaging with amyloid positron emission tomography: a systematic review and meta-analysis. Journal of Nuclear Cardiology..

[20] Falk RH, Alexander KM, Liao R, Dorbala S (2016). AL (light-chain) cardiac amyloidosis: a review of diagnosis and therapy. J Am Coll Cardiol..

[21] Maurer MS, Elliott P, Comenzo R, Semigran M, Rapezzi C (2017). Addressing common questions encountered in the diagnosis and management of cardiac amyloidosis. Circulation..

[22] Möckelind S, Axelsson J, Pilebro B, Lindqvist P, Suhr OB, Sundström T (2020). Quantification of cardiac amyloid with [^18^F]Flutemetamol in patients with V30M hereditary transthyretin amyloidosis. Amyloid..

[23] Lhommel R, Sempoux C, Ivanoiu A, Michaux L, Gerber B (2014). Is 18F-flutemetamol PET/CT able to reveal cardiac amyloidosis?. Clin Nucl Med..

[24] Kircher M, Ihne S, Brumberg J (2019). Detection of cardiac amyloidosis with 18F-Florbetaben-PET/CT in comparison to echocardiography, cardiac MRI and DPD-scintigraphy. Eur J Nucl Med Mol Imaging..

[25] Palladini G, Dispenzieri A, Gertz MA (2012). New criteria for response to treatment in immunoglobulin light chain amyloidosis based on free light chain measurement and cardiac biomarkers: impact on survival outcomes. J Clin Oncol..

[26] Martinez-Naharro A, Kotecha T, Norrington K (2019). Native T1 and extracellular volume in transthyretin amyloidosis. JACC Cardiovasc Imaging..

[27] Gillmore JD, Maurer MS, Falk RH (2016). Nonbiopsy diagnosis of cardiac transthyretin amyloidosis. Circulation..

