# Supplementary material for: 18F-flutemetamol positron emission tomography in cardiac amyloidosis
Source: J Nucl Cardiol. 2020 Oct 6;29(2):779–89. doi: 10.1007/s12350-020-02363-2 (PMC8993783; doi:10.1007/s12350-020-02363-2)
Supplement: Supplementary file 1 — Electronic supplementary material 1 (PPTX 4492 kb) [file 12350_2020_2363_MOESM1_ESM.pptx]

## Slide 1
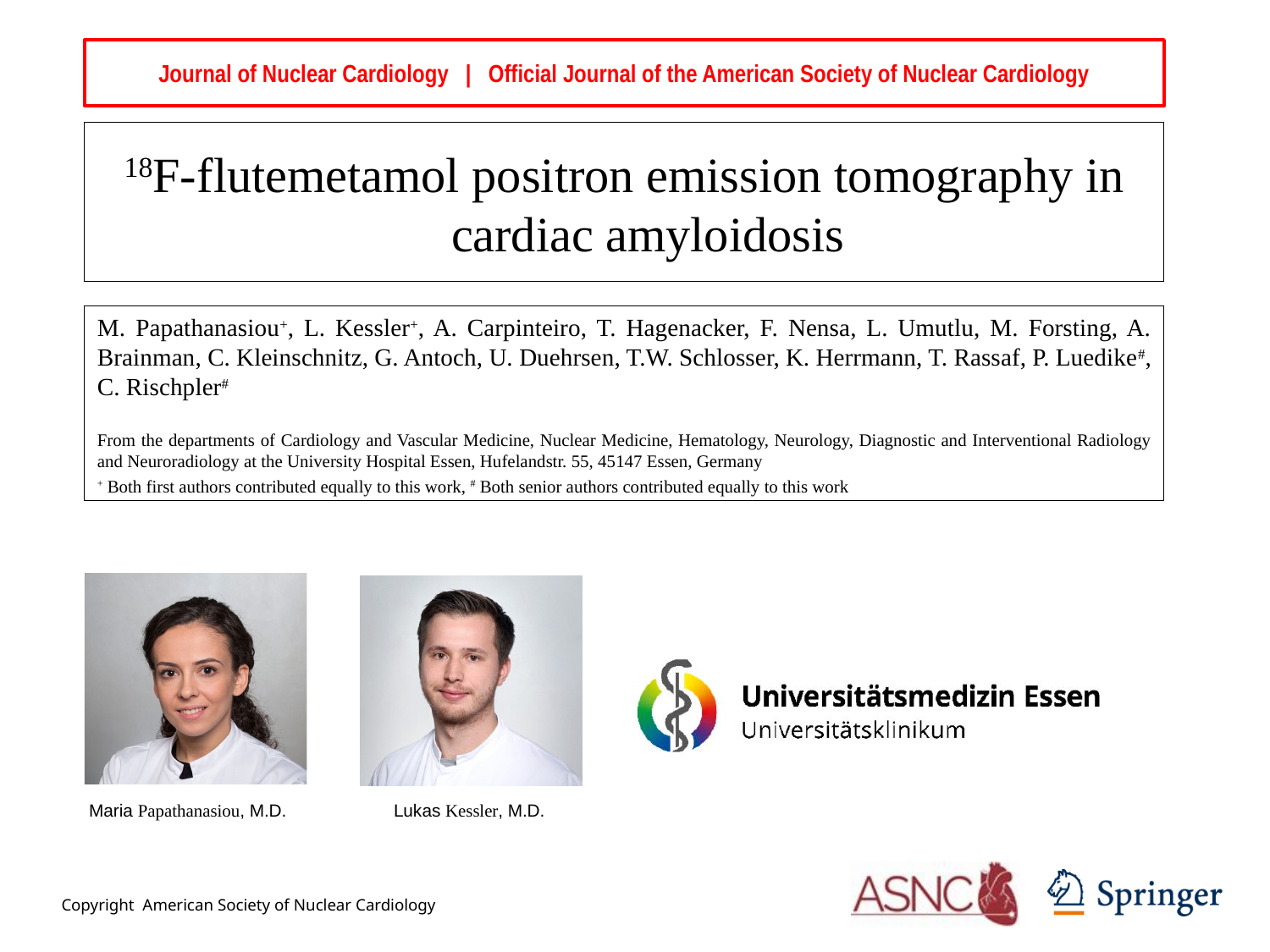

Journal of Nuclear Cardiology | Official Journal of the American Society of Nuclear Cardiology
# 18F-flutemetamol positron emission tomography in cardiac amyloidosis
M. Papathanasiou+, L. Kessler+, A. Carpinteiro, T. Hagenacker, F. Nensa, L. Umutlu, M. Forsting, A. Brainman, C. Kleinschnitz, G. Antoch, U. Duehrsen, T.W. Schlosser, K. Herrmann, T. Rassaf, P. Luedike#, C. Rischpler#
From the departments of Cardiology and Vascular Medicine, Nuclear Medicine, Hematology, Neurology, Diagnostic and Interventional Radiology and Neuroradiology at the University Hospital Essen, Hufelandstr. 55, 45147 Essen, Germany
+ Both first authors contributed equally to this work, # Both senior authors contributed equally to this work
Lukas Kessler, M.D.
Maria Papathanasiou, M.D.
Copyright American Society of Nuclear Cardiology

## Slide 2
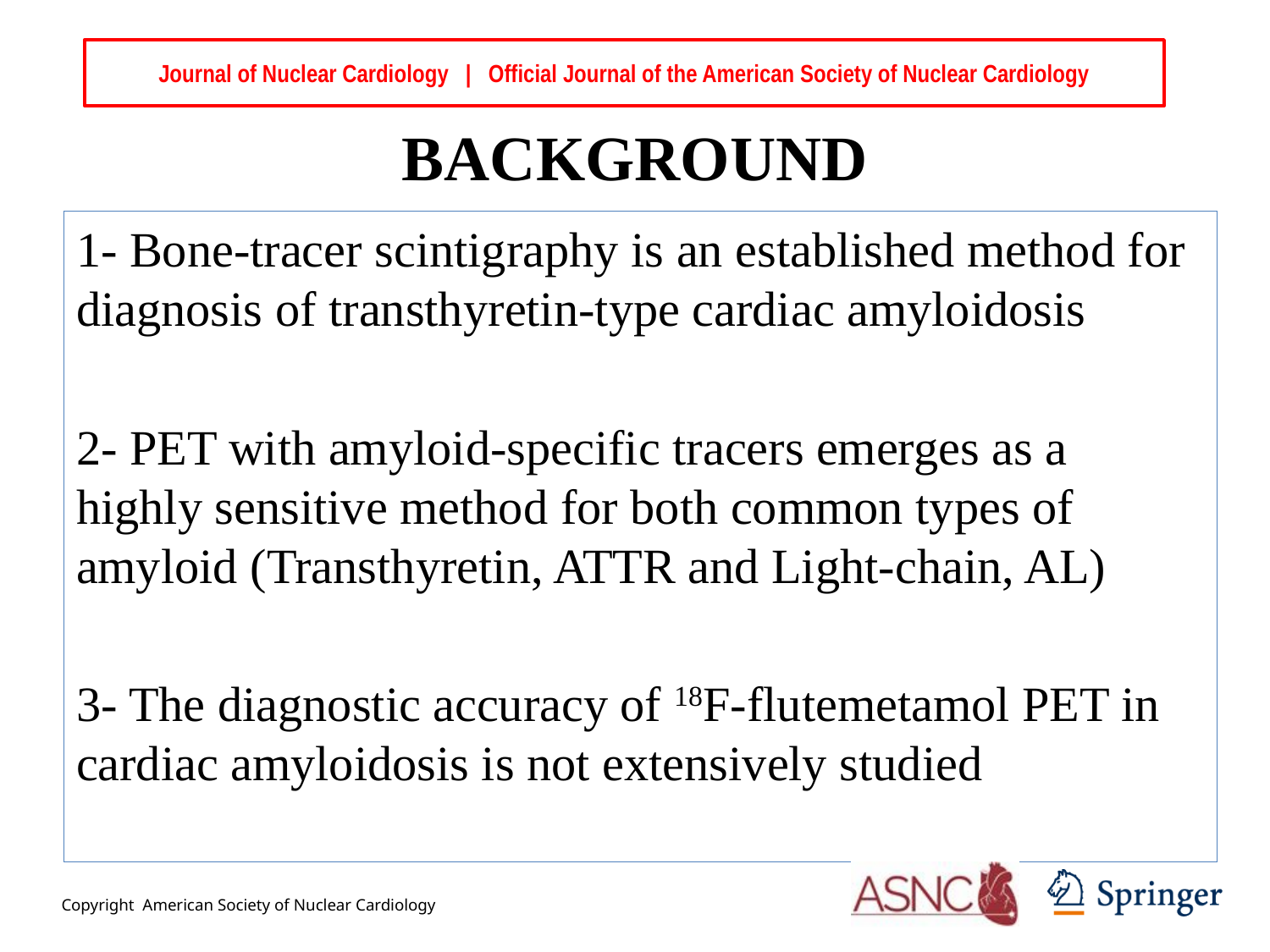

Journal of Nuclear Cardiology | Official Journal of the American Society of Nuclear Cardiology
# BACKGROUND
1- Bone-tracer scintigraphy is an established method for diagnosis of transthyretin-type cardiac amyloidosis
2- PET with amyloid-specific tracers emerges as a highly sensitive method for both common types of amyloid (Transthyretin, ATTR and Light-chain, AL)
3- The diagnostic accuracy of 18F-flutemetamol PET in cardiac amyloidosis is not extensively studied
Copyright American Society of Nuclear Cardiology

## Slide 3
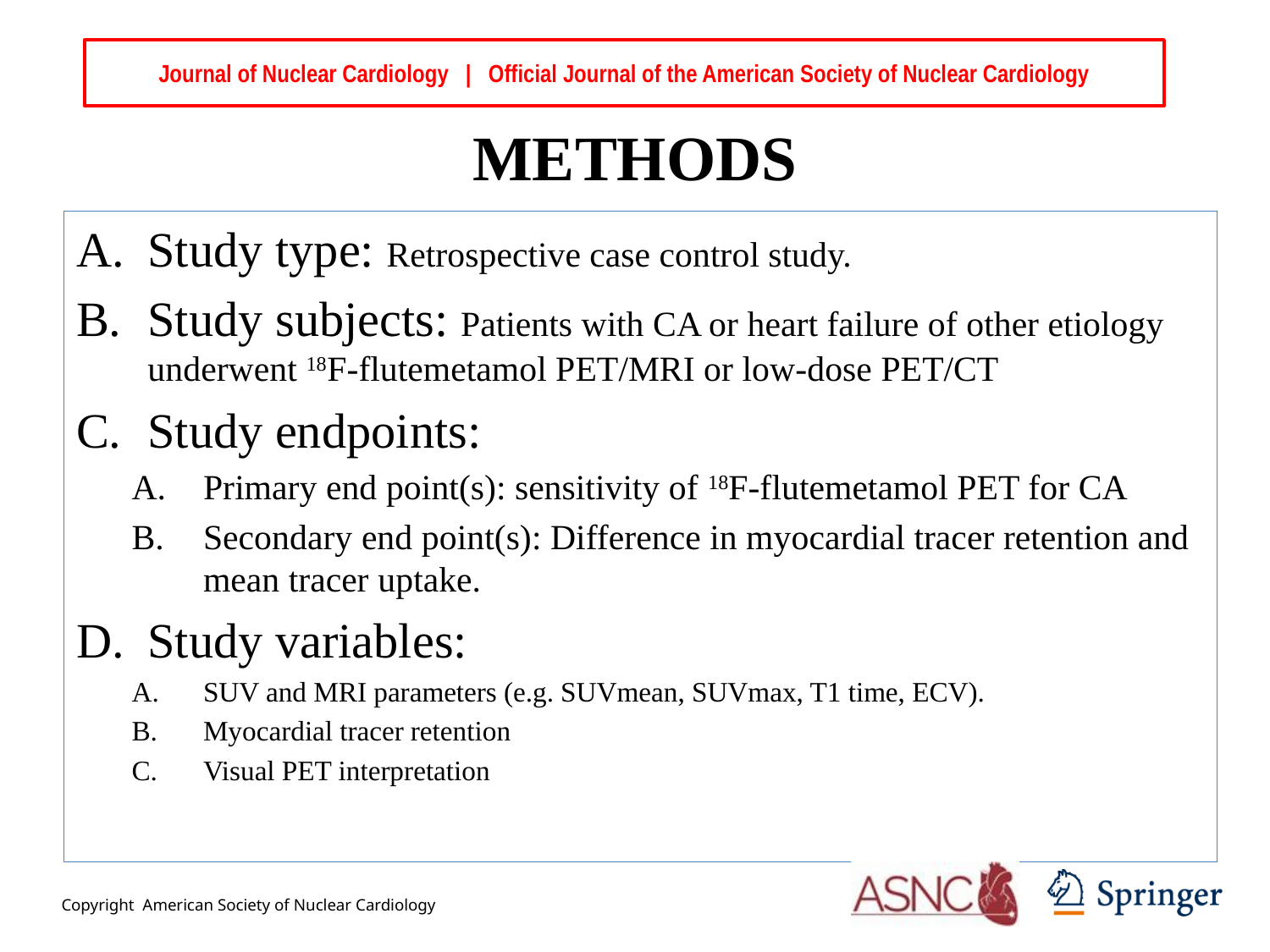

Journal of Nuclear Cardiology | Official Journal of the American Society of Nuclear Cardiology
# METHODS
Study type: Retrospective case control study.
Study subjects: Patients with CA or heart failure of other etiology underwent 18F-flutemetamol PET/MRI or low-dose PET/CT
Study endpoints:
Primary end point(s): sensitivity of 18F-flutemetamol PET for CA
Secondary end point(s): Difference in myocardial tracer retention and mean tracer uptake.
Study variables:
SUV and MRI parameters (e.g. SUVmean, SUVmax, T1 time, ECV).
Myocardial tracer retention
Visual PET interpretation
Copyright American Society of Nuclear Cardiology

## Slide 4
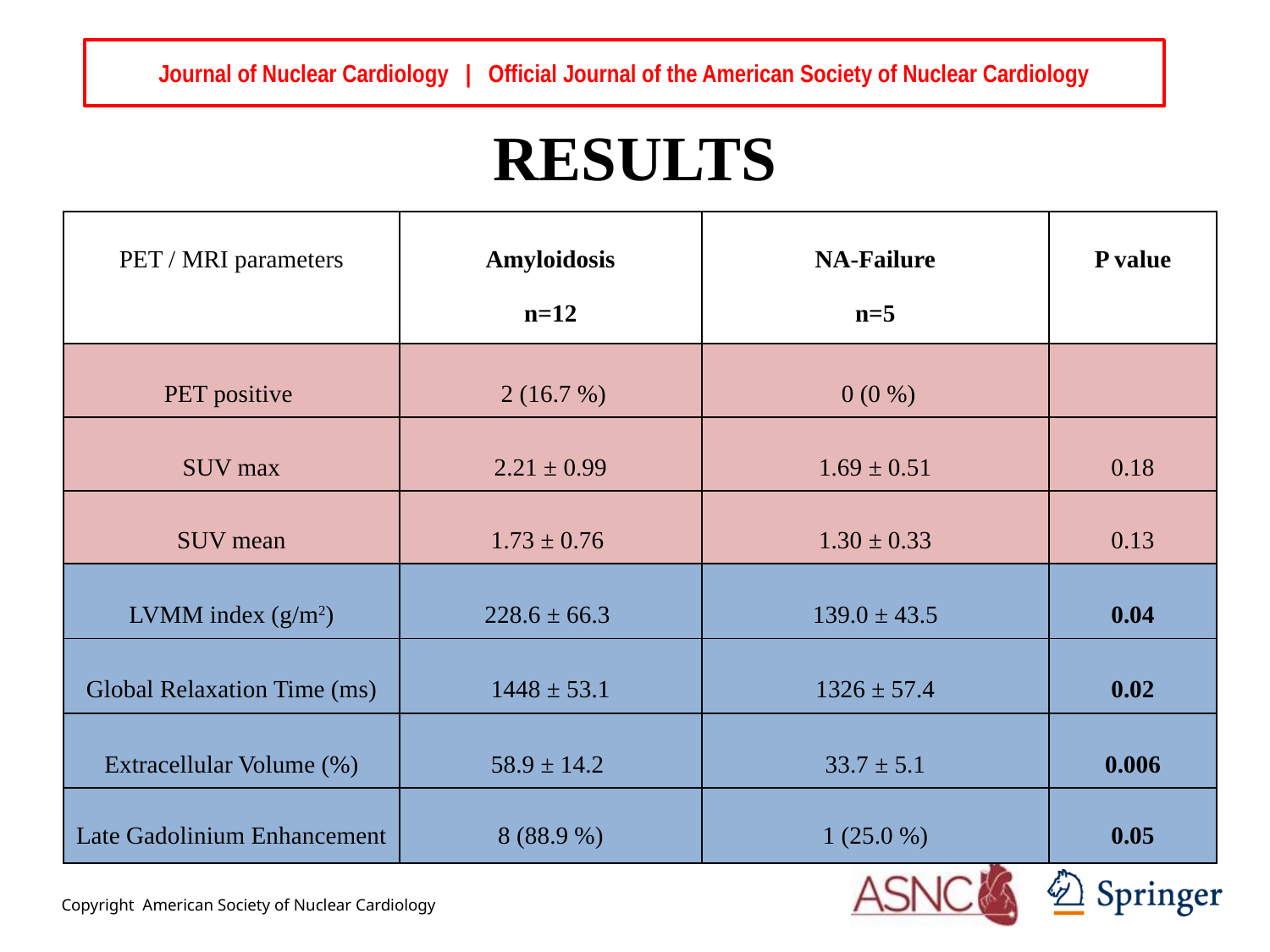

Journal of Nuclear Cardiology | Official Journal of the American Society of Nuclear Cardiology
# RESULTS
| PET / MRI parameters | Amyloidosis n=12 | NA-Failure n=5 | P value |
| --- | --- | --- | --- |
| PET positive | 2 (16.7 %) | 0 (0 %) | |
| SUV max | 2.21 ± 0.99 | 1.69 ± 0.51 | 0.18 |
| SUV mean | 1.73 ± 0.76 | 1.30 ± 0.33 | 0.13 |
| LVMM index (g/m2) | 228.6 ± 66.3 | 139.0 ± 43.5 | 0.04 |
| Global Relaxation Time (ms) | 1448 ± 53.1 | 1326 ± 57.4 | 0.02 |
| Extracellular Volume (%) | 58.9 ± 14.2 | 33.7 ± 5.1 | 0.006 |
| Late Gadolinium Enhancement | 8 (88.9 %) | 1 (25.0 %) | 0.05 |
Copyright American Society of Nuclear Cardiology

## Slide 5
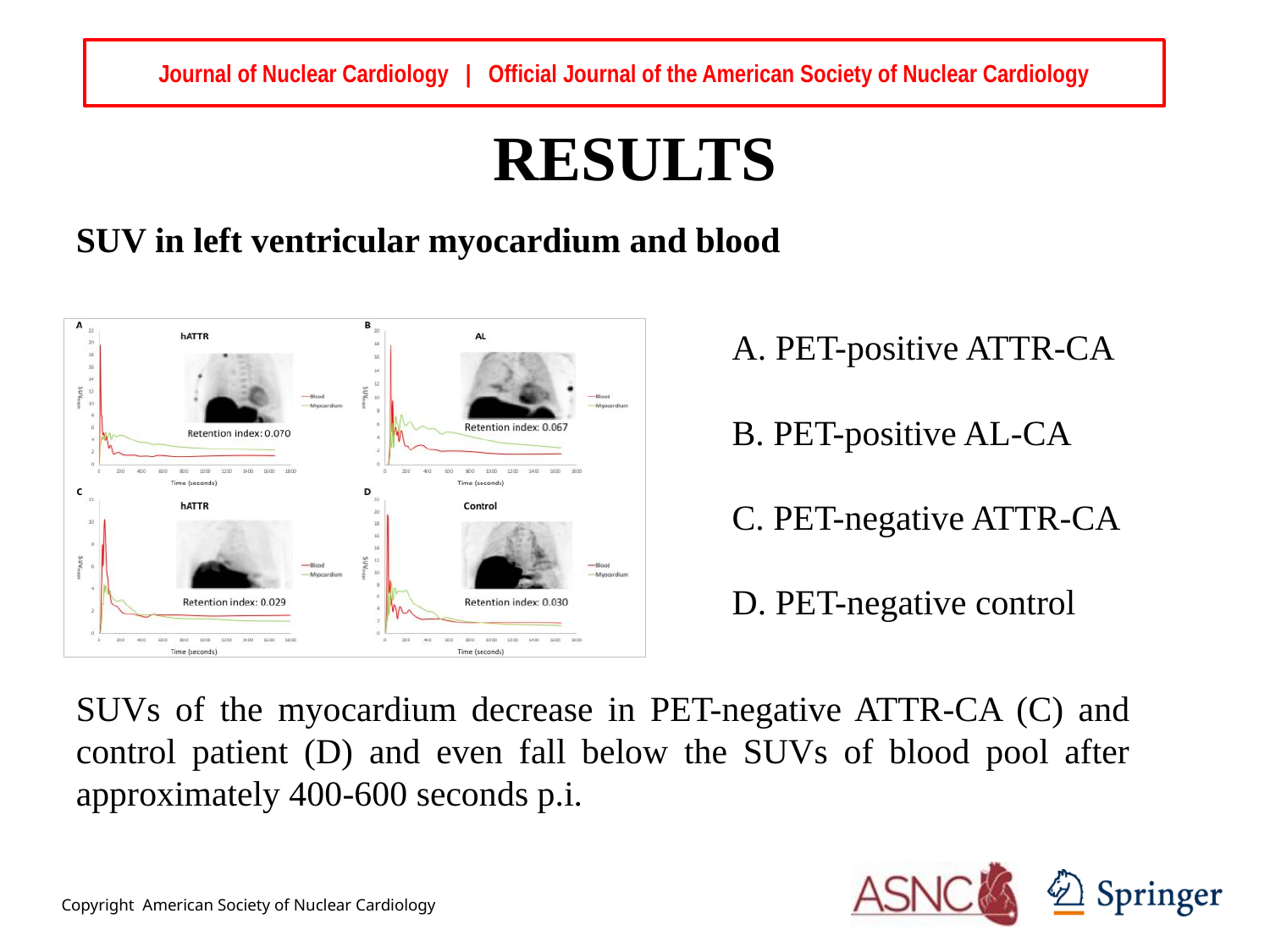

Journal of Nuclear Cardiology | Official Journal of the American Society of Nuclear Cardiology
# RESULTS
SUV in left ventricular myocardium and blood
A. PET-positive ATTR-CA
B. PET-positive AL-CA
C. PET-negative ATTR-CA
D. PET-negative control
SUVs of the myocardium decrease in PET-negative ATTR-CA (C) and control patient (D) and even fall below the SUVs of blood pool after approximately 400-600 seconds p.i.
Copyright American Society of Nuclear Cardiology

## Slide 6
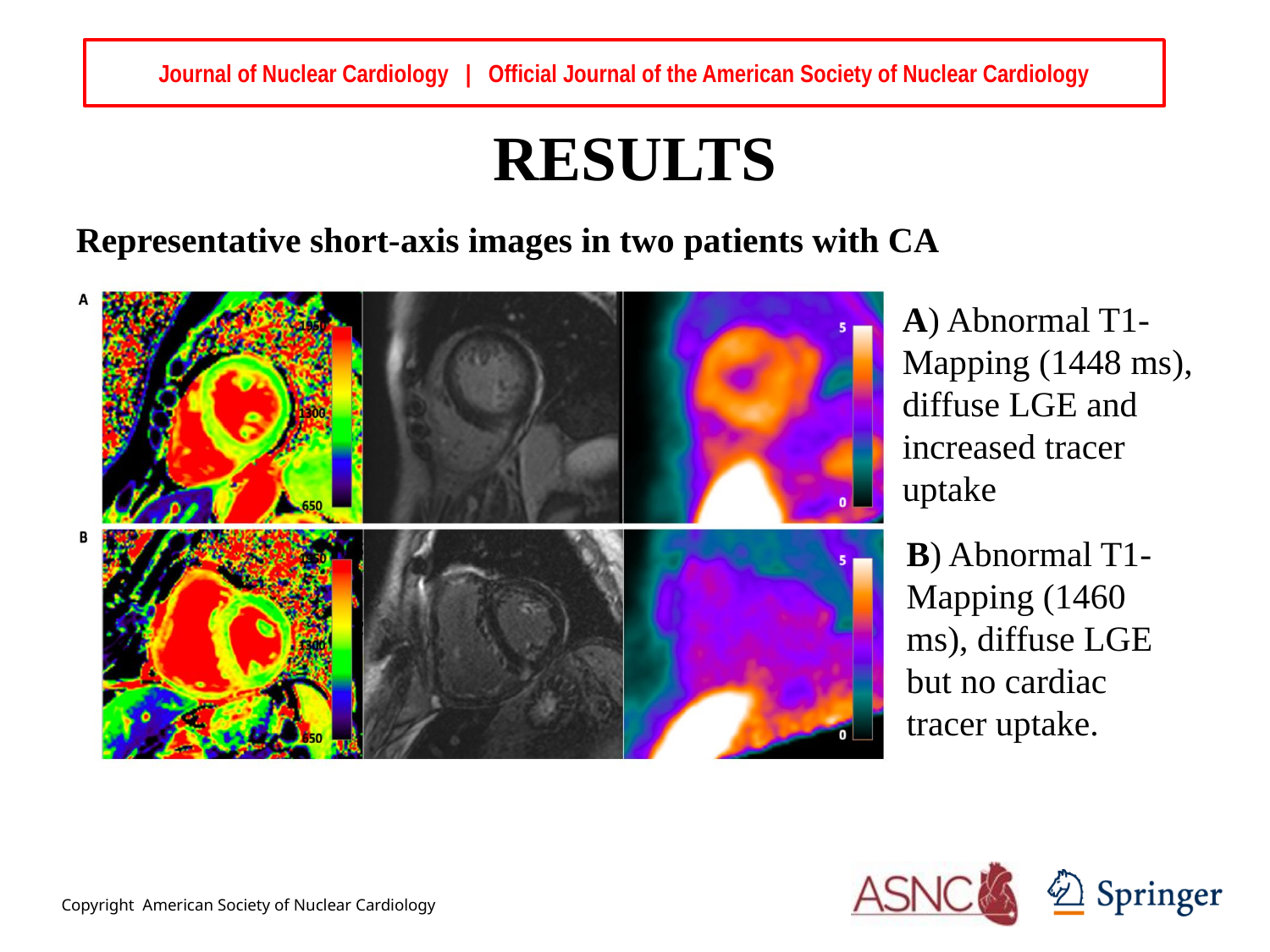

Journal of Nuclear Cardiology | Official Journal of the American Society of Nuclear Cardiology
# RESULTS
Representative short-axis images in two patients with CA
A) Abnormal T1-Mapping (1448 ms), diffuse LGE and increased tracer uptake
B) Abnormal T1-Mapping (1460 ms), diffuse LGE but no cardiac tracer uptake.
Copyright American Society of Nuclear Cardiology

## Slide 7
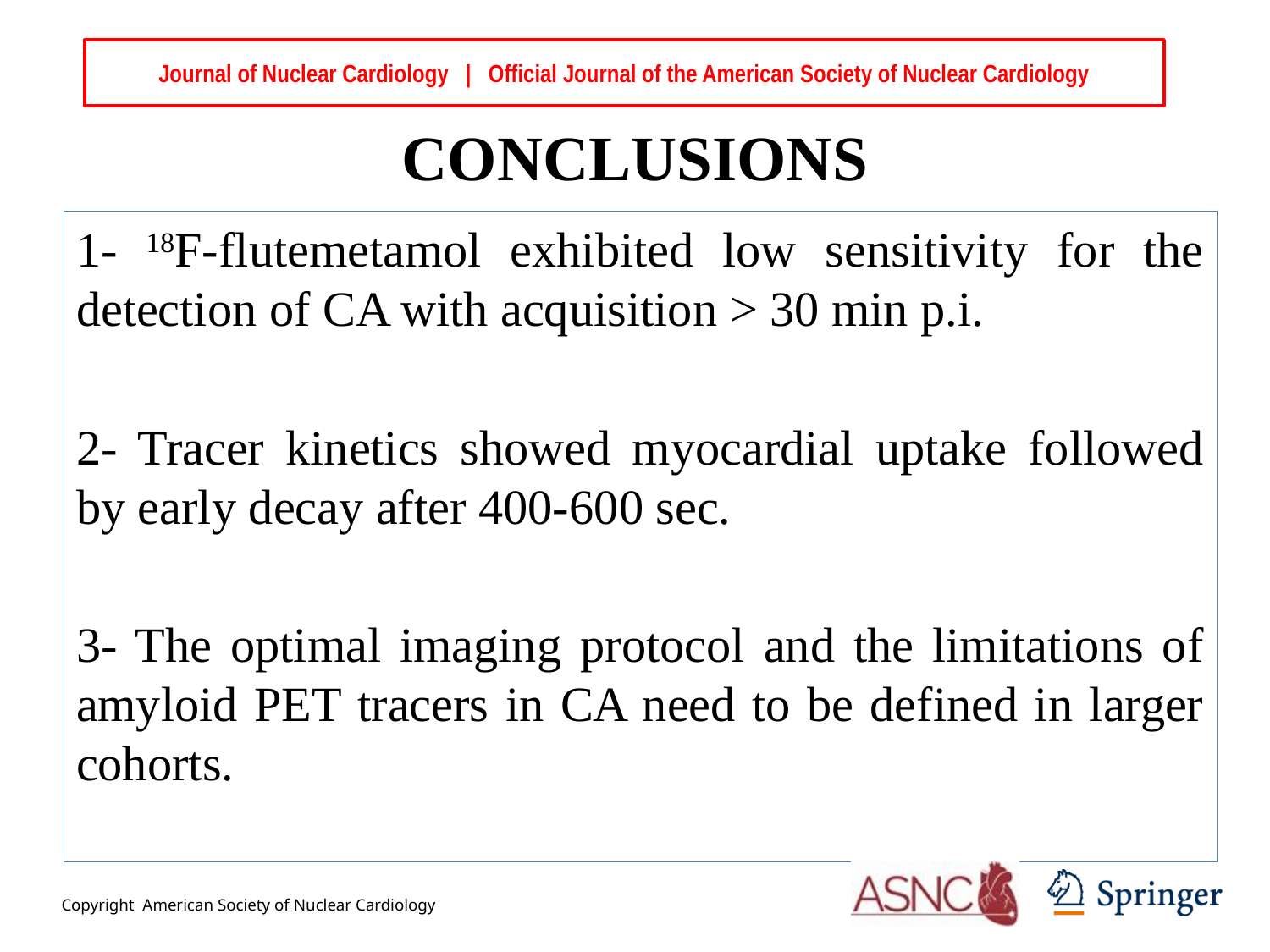

Journal of Nuclear Cardiology | Official Journal of the American Society of Nuclear Cardiology
# CONCLUSIONS
1- 18F-flutemetamol exhibited low sensitivity for the detection of CA with acquisition > 30 min p.i.
2- Tracer kinetics showed myocardial uptake followed by early decay after 400-600 sec.
3- The optimal imaging protocol and the limitations of amyloid PET tracers in CA need to be defined in larger cohorts.
Copyright American Society of Nuclear Cardiology
